# The ratio of LDL-C to HDL-C can effectively predict the prognosis of IgA nephropathy

**DOI:** 10.3389/fmed.2026.1762427

**Published:** 2026-04-13

**Authors:** Ya Hu, Wanwan Zhao, Dongdong Mei, Ganxiu Li, Sishi Lin, Chaosheng Chen

**Affiliations:** 1 Department of Nephrology, Fuyang People’s Hospital Affiliated to Anhui Medical University, Fuyang, Anhui, China; 2 Department of Nephrology, The First Affiliated Hospital of Wenzhou Medical University, Wenzhou, Zhejiang, China

**Keywords:** ESRD, IgA nephropathy, immunoglobulin, LDL-C/HDL-C, lipid metabolism, prognosis

## Abstract

**Background:**

Abnormal lipid metabolism affects IgA nephropathy (IgAN) prognosis, but the prognostic value of LDL-C/HDL-C ratio in IgAN remains unclear.

**Methods:**

A bicenter retrospective cohort study enrolled 1,405 IgAN patients with a median follow-up of 50.1 months. Patients were divided into four quartiles based on LDL-C/HDL-C ratio (Q1: lowest quartile, Q4: highest quartile). Kaplan–Meier, Cox regression, and restricted cubic spline (RCS) analyses were used to explore the association between LDL-C/HDL-C ratio and end-stage renal disease (ESRD) risk.

**Results:**

ESRD occurred in 110 patients (8.45%). The ESRD-free survival rate decreased significantly with increasing LDL-C/HDL-C quartiles (log-rank *p* < 0.001). Multivariate Cox regression analysis demonstrated that, compared with the first quartile (Q1, reference), the hazard ratios for Q2, Q3, and Q4 were 2.542 (95% CI: 0.679–9.523, *p* = 0.166), 2.499 (95% CI: 0.707–8.830, *p* = 0.155), and 6.384 (95% CI: 1.840–22.154, *p* = 0.003), respectively. Each 1-unit increase in the LDL-C/HDL-C ratio was independently associated with a 45.2% elevated risk (HR = 1.452, 95% CI: 1.159–1.818, *p* = 0.001). Restricted cubic splines (RCS) analysis confirmed a linear dose–response relationship (overall *p* = 0.0196, nonlinear *p* = 0.2199). Subgroup analysis revealed that the association was amplified in patients with elevated serum creatinine (sCr), hypertension, hyperuricemia, M1, and T2 (all *p* < 0.05). However, the LDL-C/HDL-C ratio exhibited no correlation with renal IgA deposition intensity (*r* = 0.013, *p* = 0.624).

**Conclusion:**

Elevated LDL-C/HDL-C ratio constitutes an independent risk factor for end-stage renal disease (ESRD) in patients with IgA nephropathy (IgAN), demonstrating a linear dose–response relationship and serving as a reliable prognostic biomarker independent of IgA deposition.

## 1 Introduction

IgA nephropathy (IgAN) is the most prevalent primary glomerular disease worldwide, characterized by dominant mesangial IgA deposition and a high risk of progression to chronic kidney disease (CKD) and end-stage renal disease (ESRD) (1). Approximately 15 to 40% of IgAN patients progress to ESRD within a decade of diagnosis, and Chinese epidemiological data show that IgAN accounts for 43.5 to 58.2% of primary glomerular diseases diagnosed by renal biopsy—with its actual prevalence likely underestimated due to subclinical cases (1, 2). Given the frequently chronic trajectory of IgA nephropathy, focusing on prognosis and implementing early intervention become critically important.

Lipid metabolism abnormalities are well-recognized risk factors for kidney function decline and cardiovascular complications in CKD patients (3). Low-density lipoprotein cholesterol (LDL-C) is the primary target of lipid-lowering interventions in CKD (4), and elevated LDL-C levels in IgAN patients are associated with more severe clinical features, interstitial renal pathology, and increased ESRD risk (5). High-density lipoprotein cholesterol (HDL-C) exerts anti-inflammatory, antioxidant, and vasoprotective effects (6), and low HDL-C concentrations are the only lipid alteration linked to kidney disease progression in mild to moderate CKD (7). Notably, LDL and HDL often exert opposite effects in renal and cardiovascular pathology, and not all lipid abnormalities involve simultaneous LDL elevation and HDL reduction; thus, the combined evaluation of LDL and HDL is more comprehensive for predicting CKD prognosis than single lipid indicators (8).

The LDL-C/HDL-C ratio has been shown to have better predictive value for coronary atherosclerotic heart disease and carotid intima-media thickness than single lipid indicators (8, 9), and an elevated LDL-C/HDL-C ratio is a risk factor for renal dysfunction in the general population (10). However, its prognostic value in IgAN remains unclear, which is the research gap addressed in this study.

Notably, this study is the first bicenter cohort study to confirm the linear dose–response relationship between LDL-C/HDL-C ratio and ESRD risk in IgAN patients via RCS analysis, and further identifies high-risk subgroups (elevated sCr, hypertension, hyperuricemia, M1, T2) where the prognostic value is amplified. Moreover, we demonstrate that the prognostic value of LDL-C/HDL-C ratio is independent of renal IgA deposition, suggesting lipid metabolism disorder is an independent pathological process in IgAN progression, which has not been fully elucidated in previous studies.

## 2 Materials and methods

### 2.1 Study population

We initially enrolled 2,567 patients diagnosed with IgAN via renal biopsy at Fuyang People’s Hospital Affiliated to Anhui Medical University and The First Affiliated Hospital of Wenzhou Medical University between January 2011 and January 2024. Exclusion criteria were: (1) other primary or secondary glomerular diseases; (2) secondary IgAN-related conditions (systemic lupus erythematosus, allergic purpura, viral hepatitis B); (3) incomplete baseline clinical and pathological data; (4) follow-up duration <6 months. Finally, 1,405 patients with primary IgAN were included in the analysis. The study strictly adhered to the Declaration of Helsinki and was approved by the Hospital Ethics Committee of Fuyang People’s Hospital (approval number: FYHR2024-062). Due to the retrospective design and use of anonymized data, written informed consent from patients was waived by the ethics committees of all participating institutions.

### 2.2 Clinical and pathological data collection

Baseline clinical data—including age, gender, systolic blood pressure (SBP), diastolic blood pressure (DBP), and key laboratory indicators [serum neutrophil count (sNC), platelet count (PLT), serum lymphocyte count (sLC), hemoglobin, serum creatinine (sCr), uric acid, albumin (Alb), triglycerides (TG), total cholesterol (TC), LDL-C, HDL-C, fibrinogen (Fib), and 24-h urine protein]—were meticulously collected from comprehensive electronic medical records. The LDL-C/HDL-C ratio was derived by dividing the serum LDL-C concentration by the HDL-C concentration. Furthermore, eGFR was calculated using the CKD-EPI (Chronic Kidney Disease Epidemiology Collaboration) formula (11), the gold standard widely recognized for eGFR estimation in both clinical practice and research.

Renal biopsy was performed by professional nephrologists using standard techniques, and pathological findings were assessed by expert renal pathologists according to the 2016 IgAN Oxford Classification (9), including intracapillary cytosis (E0/1), mesangial hypercellularity (M0/1), tubular atrophy and interstitial fibrosis (T0/1/2), segmental glomerulosclerosis (S0/1), and crescents (C0/1). Immunofluorescence (IF) was used to detect the deposition intensity of renal immunoglobulin A/G/M (IgA/G/M), classified as: − (negative), + (mild), ++ (moderate), +++ (severe), +++++ (massive) (12). We also recorded the primary treatment regimens during hospitalization and follow-up, including glucocorticoids, immunosuppressants, and renin-angiotensin-aldosterone system (RAAS) inhibitors (ACEI/ARB).

### 2.3 Outcome definition

The primary study outcome was end-stage renal disease (ESRD), defined as the initiation of renal replacement therapy (hemodialysis, peritoneal dialysis, or kidney transplantation) or an estimated glomerular filtration rate (eGFR) <15 mL/min/1.73 m^2^ (1).

### 2.4 Statistical analysis

All statistical analyses were conducted using R software (version 4.2.1). A two-sided *p*-value <0.05 was considered statistically significant.

*Descriptive statistics*: Normally distributed continuous variables are presented as mean ± standard deviation, with group comparisons via independent samples *t*-tests or one-way analysis of variance (ANOVA). Non-normally distributed continuous variables are expressed as median [interquartile range (IQR)], with group comparisons via Wilcoxon rank-sum test or Kruskal–Wallis test. Categorical variables are reported as counts (percentages), with group comparisons via *χ*^2^ test or Fisher’s exact test (as appropriate).

*Correlation analysis*: Spearman correlation analysis was employed to examine the relationship between LDL-C/HDL-C ratio and other clinical indicators (13).

*Survival analysis*: Kaplan–Meier survival curves were utilized to plot cumulative ESRD-free survival rates across LDL-C/HDL-C quartiles, with the log-rank test used to compare survival differences among groups (14).

*Cox regression analysis*: Univariate Cox proportional hazards regression models screened potential risk factors for ESRD (variables with *p* < 0.1 were incorporated into the multivariate model). Multivariate Cox proportional hazards regression models analyzed the independent association between LDL-C/HDL-C ratio (as quartile groups and continuous variable) and ESRD risk, adjusted for confounders (gender, SBP, DBP, sCr, urine protein, uric acid, Alb, hemoglobin, TG, TC, renal pathological classification [M, T], immunoglobulin deposition intensity, treatment regimens). Results are expressed as hazard ratios (HR) with 95% confidence intervals (95% CI) (15).

*Restricted cubic spline (RCS) analysis*: A 3-knot multivariate-adjusted RCS analysis (knots at 10th, 50th, 90th percentiles of LDL-C/HDL-C ratio) tested the dose–response relationship, using the median LDL-C/HDL-C ratio as the reference. Overall association *p*-value and nonlinear trend *p*-value were calculated to assess linearity (16).

*Subgroup analysis*: Stratified subgroup analyses were conducted based on baseline clinical and pathological characteristics (age, gender, hypertension, hyperuricemia, sCr level, M classification, T classification). Interaction *p*-values were computed the consistency of the association between LDL-C/HDL-C ratio and ESRD risk across subgroups (17).

## 3 Results

### 3.1 Baseline characteristics

**Figure 1:**
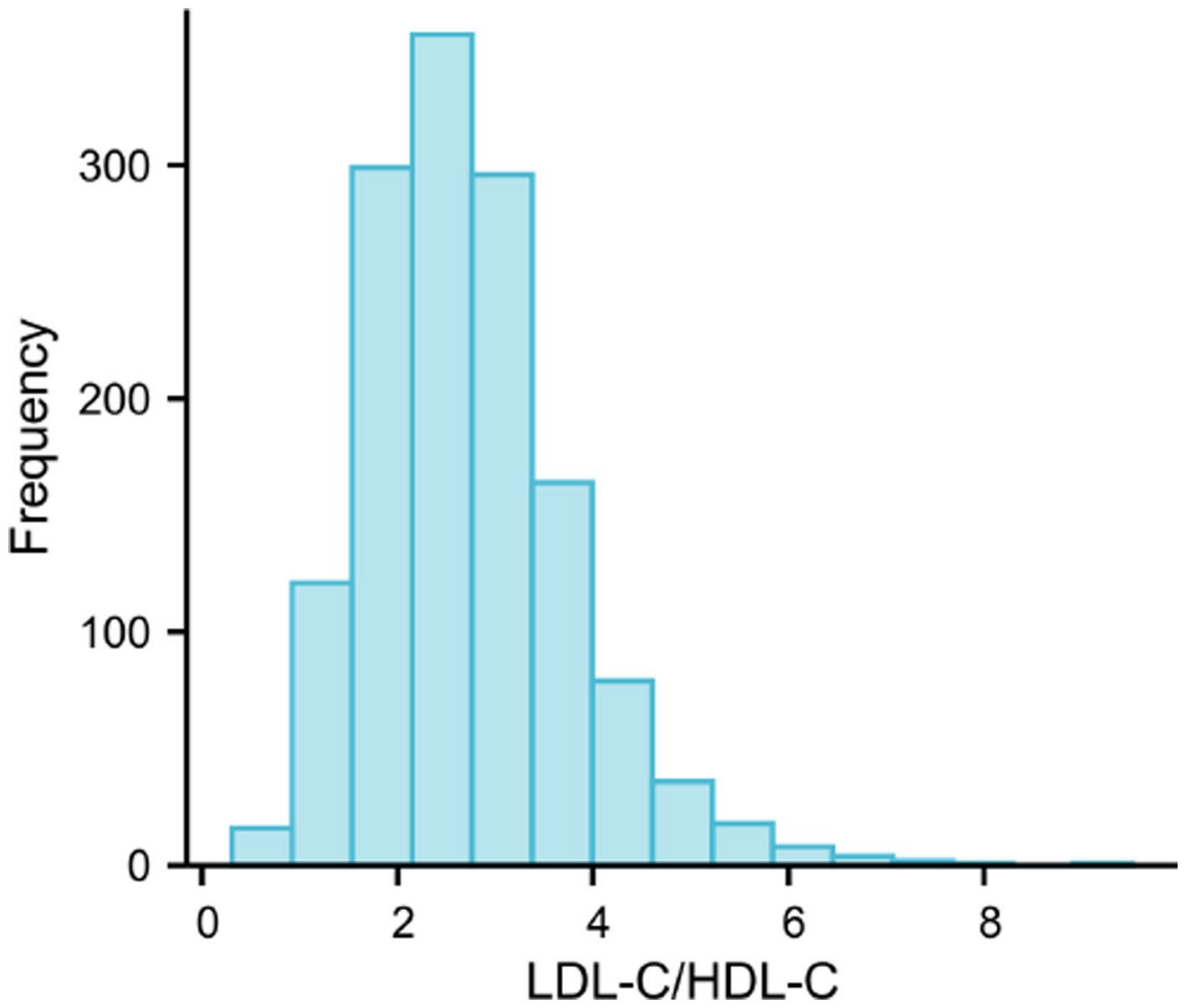
Histogram showing the distribution of LDL-C/HDL-C ratio in 1405 primary IgA nephropathy patients included in the study; LDL-C, low-density lipoprotein cholesterol; HDL-C, high-density lipoprotein cholesterol.

The 1,405 enrolled patients had a mean age of 38.7 years and a median follow-up of 50.1 months (range across quartiles: 44.7–50.8 months; *p* = 0.410 for interquartile difference) (Table 1). Patients were stratified into four quartiles based on baseline LDL-C/HDL-C ratio: Q1 (lowest quartile, *n* = 350), Q2 (*n* = 350), Q3 (*n* = 354), and Q4 (highest quartile, *n* = 351). Patients in Q2–Q4 of LDL-C/HDL-C ratio were older, had a higher male proportion, and presented with elevated levels of triglycerides, total cholesterol, hemoglobin, fibrinogen, sLC, sNC, creatinine, uric acid, LDL-C, and urinary protein—concomitant with reduced albumin and eGFR (all *p* < 0.001). Pathologically, these patients had more pronounced mesangial cell proliferation and tubulointerstitial fibrosis, and immunosuppressive agents were more frequently utilized (*p* < 0.001) (see Figure 1).

**Table 1:** Clinical baseline characteristics of patients grouped according to LDL-C/HDL-C quartiles.

Characteristics	Q1 (*n* = 350)	Q2 (*n* = 350)	Q3 (*n* = 354)	Q4 (*n* = 351)	*p*-value
Age (years)	36 (28, 46)	38 (31, 47)	41 (31, 49)	40 (31, 49)	0.002
Male, *n* (%)	111 (31.7)	134 (38.3)	166 (46.9)	196 (55.8)	<0.001
SBP (mmHg)	116.95 (107.68, 130.00)	120.50 (110.57, 134.00)	122.58 (115.00, 134.06)	123.76 (116.25, 135.33)	<0.001
DBP (mmHg)	72.00 (65.16, 80.00)	75.00 (67.82, 83.86)	76.93 (70.00, 82.00)	77.67 (70.50, 85.00)	<0.001
Pathological features					
M1, *n* (%)	106 (30.3)	125 (35.7)	133 (37.6)	119 (33.9)	0.167
E1, *n* (%)	107 (30.6)	131 (37.4)	118 (33.3)	120 (34.2)	0.087
S1, *n* (%)	190 (54.3)	199 (56.9)	206 (58.2)	190 (54.1)	0.742
T category, *n* (%)					<0.001
T0 (no atrophy/fibrosis)	277 (79.1)	257 (73.4)	253 (71.5)	227 (64.7)	
T1 (mild–moderate)	50 (14.3)	71 (20.3)	77 (21.8)	88 (25.1)	
T2 (severe)	23 (6.6)	22 (6.3)	24 (6.8)	36 (10.3)	
C1, *n* (%)	174 (49.7)	179 (51.1)	186 (52.5)	191 (54.4)	0.105
Renal Ig deposition, *n* (%)					
IgA deposition					0.369
+	87 (24.9)	89 (25.4)	104 (29.4)	109 (31.1)	
++	22 (6.3)	24 (6.9)	26 (7.3)	23 (6.6)	
+++	230 (65.7)	221 (63.1)	208 (58.8)	208 (59.3)	
+++++	7 (2.0)	14 (4.0)	13 (3.7)	7 (2.0)	
IgG deposition					<0.001
+	15 (4.3)	18 (5.1)	11 (3.1)	16 (4.6)	
++	17 (4.9)	5 (1.4)	15 (4.2)	9 (2.6)	
+++	1 (0.3)	0 (0.0)	9 (2.5)	1 (0.3)	
IgM deposition					0.743
+	97 (27.7)	88 (25.1)	89 (25.1)	90 (25.6)	
++	47 (13.4)	40 (11.4)	48 (13.6)	41 (11.7)	
+++	9 (2.6)	8 (2.3)	7 (2.0)	3 (0.9)	
Clinical laboratory indices					
Hemoglobin (g/L)	124 (114, 135)	124 (113, 139)	128 (114, 141)	131 (117, 142.75)	<0.001
TG (mmol/L)	4.14 (3.62, 4.81)	4.63 (4.07, 5.39)	5.12 (4.52, 5.79)	5.82 (5.12, 6.76)	<0.001
TC (mmol/L)	1.24 (0.91, 2.27)	1.59 (1.08, 2.56)	1.86 (1.41, 3.02)	2.09 (1.54, 3.00)	<0.001
HDL-C (mmol/L)	1.31 (1.11, 1.51)	1.11 (0.98, 1.30)	1.02 (0.91, 1.17)	0.92 (0.81, 1.06)	<0.001
LDL-C (mmol/L)	2.05 (1.71, 2.42)	2.56 (2.28, 2.97)	3.01 (2.67, 3.41)	3.65 (3.19, 4.29)	<0.001
Fibrinogen (g/L)	3.14 (2.65, 3.69)	3.29 (2.79, 3.78)	3.45 (2.97, 4.05)	3.89 (3.16, 4.61)	<0.001
Alb (g/L)	37.8 (34.4, 41.0)	37.0 (33.8, 40.2)	36.9 (33.5, 40.0)	35.8 (30.9, 39.3)	<0.001
sCr (μmol/L)	74 (59, 98)	84.65 (65, 111)	89.75 (68.17, 112.78)	97 (74.2, 135.5)	<0.001
UA (μmol/L)	327 (275, 396)	355 (294, 430.69)	373.5 (308, 439.53)	402 (339.5, 471.5)	<0.001
sLC (×10^9^/L)	1.93 (1.59, 2.47)	2.10 (1.67, 2.58)	2.09 (1.66, 2.69)	2.21 (1.80, 2.86)	<0.001
sNC (×10^9^/L)	3.34 (2.37, 4.67)	3.63 (2.63, 4.80)	3.55 (2.57, 4.67)	3.90 (3.00, 4.96)	<0.001
Platelet count (×10^9^/L)	219 (184.25, 263.75)	241 (203.5, 280.75)	242 (196, 284)	248 (208, 290.5)	<0.001
Proteinuria (g/24 h)	0.97 (0.54, 1.86)	1.23 (0.71, 2.35)	1.34 (0.74, 2.65)	2.06 (0.97, 3.64)	<0.001
eGFR (mL/min/1.73m^2^)	97.39 (74.72, 117.68)	88.94 (64.04, 111.58)	86.22 (62.29, 108.18)	76.23 (53.22, 103.32)	<0.001
Treatment, *n* (%)					
Glucocorticoid use	183 (52.3)	187 (53.4)	187 (52.8)	215 (61.3)	0.055
ACEI/ARB use	307 (87.7)	326 (93.1)	322 (90.9)	311 (88.6)	0.070
Immunosuppressant use	98 (28.0)	92 (26.3)	103 (29.1)	138 (39.3)	<0.001
Follow-up					
ESRD endpoint, *n* (%)	8 (2.3)	22 (6.3)	35 (9.9)	45 (12.8)	<0.001
Follow-up duration (months)	49.12 (25.58, 79.16)	44.70 (26.29, 76.18)	50.88 (29.53, 84.00)	48.00 (28.42, 76.95)	0.410

Data are presented as median [interquartile range (IQR)] for non-normally distributed continuous variables, or *n* (%) for categorical variables. Alb, albumin; ACEI/ARB, angiotensin-converting enzyme inhibitor/angiotensin II receptor blocker; eGFR, estimated glomerular filtration rate; HDL-C, high-density lipoprotein cholesterol; LDL-C, low-density lipoprotein cholesterol; sLC, serum lymphocyte count; sNC, serum neutrophil count; UA, uric acid; sCr, serum creatinine; TG, triglycerides; TC, total cholesterol. Oxford classification: M1, mesangial hypercellularity; E1, intracapillary cytosis; S1, segmental glomerulosclerosis; T0/T1/T2, tubular atrophy/interstitial fibrosis (no/mild–moderate/severe); C1, crescents. Two-sided *p*-value <0.05 was considered statistically significant.

**Figure 2:**
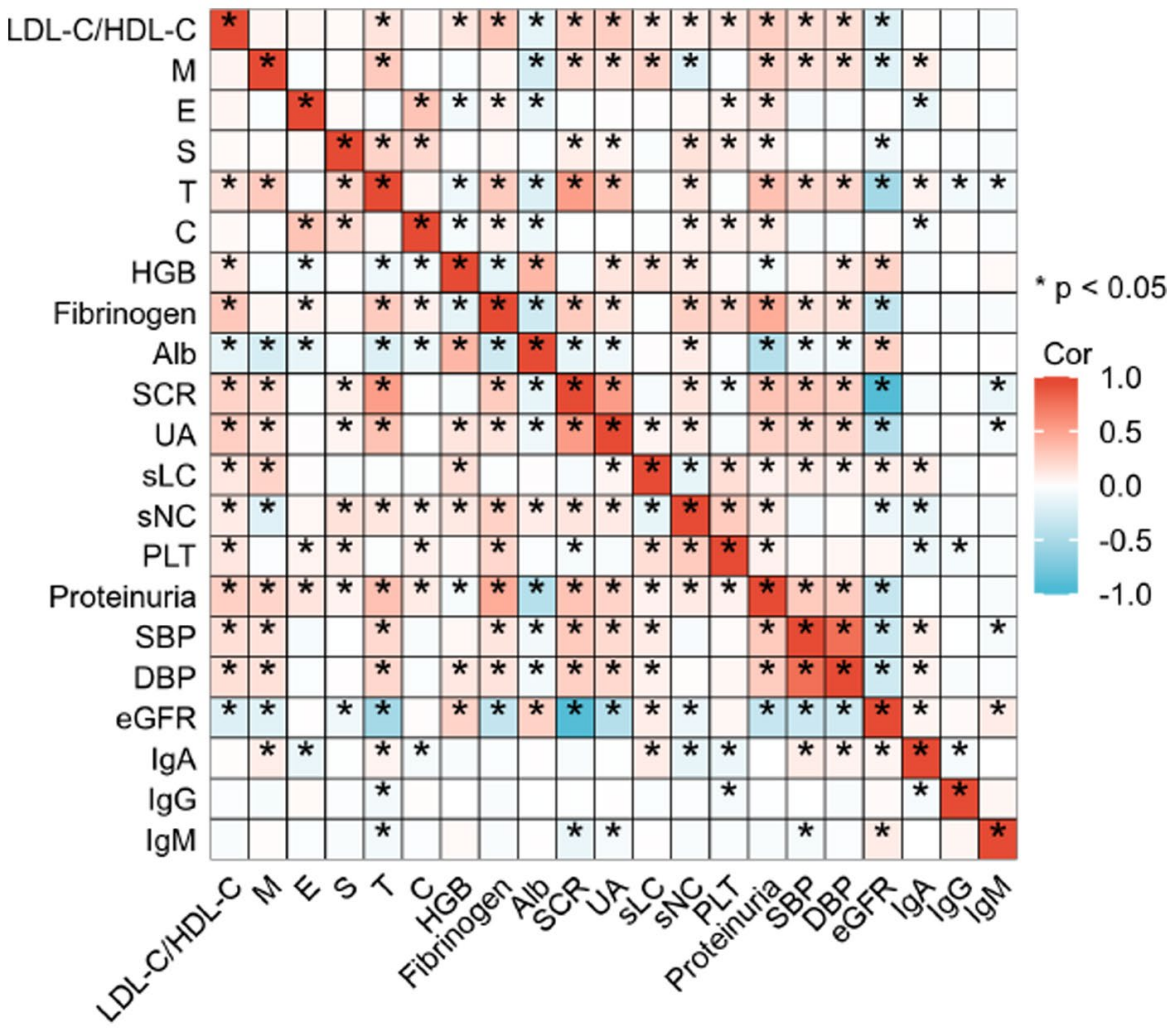
Spearman correlation heatmap depicting the association between baseline LDL-C/HDL-C ratio and clinical/pathological indicators in IgAN patients. Vibrant red signifies a positive correlation, deep blue denotes a negative correlation, and the depth of hue reflects the strength of the relationship. *p < 0.05. Abbreviations as in Table 1.

No significant differences in renal IgA deposition intensity (*p* = 0.369) or IgM deposition intensity (*p* = 0.743) were observed across quartiles. While renal IgG deposition intensity varied significantly among quartiles (*p* < 0.001), no monotonic trend was identified. Spearman correlation analysis confirmed that baseline LDL-C/HDL-C ratio was inversely correlated with albumin and eGFR, and directly correlated with creatinine, proteinuria, leukocyte count, and neutrophil count (Figure 2). Notably, the LDL-C/HDL-C ratio showed no correlation with renal IgA deposition (*r* = 0.013, *p* = 0.624) or IgM deposition (*r* = −0.022, *p* = 0.405). Its weak association with IgG deposition was non-significant (*r* = −0.039, *p* = 0.145) (Table 1, Figure 2).

### 3.2 Association between LDL-C/HDL-C ratio and ESRD risk

**Figure 3:**
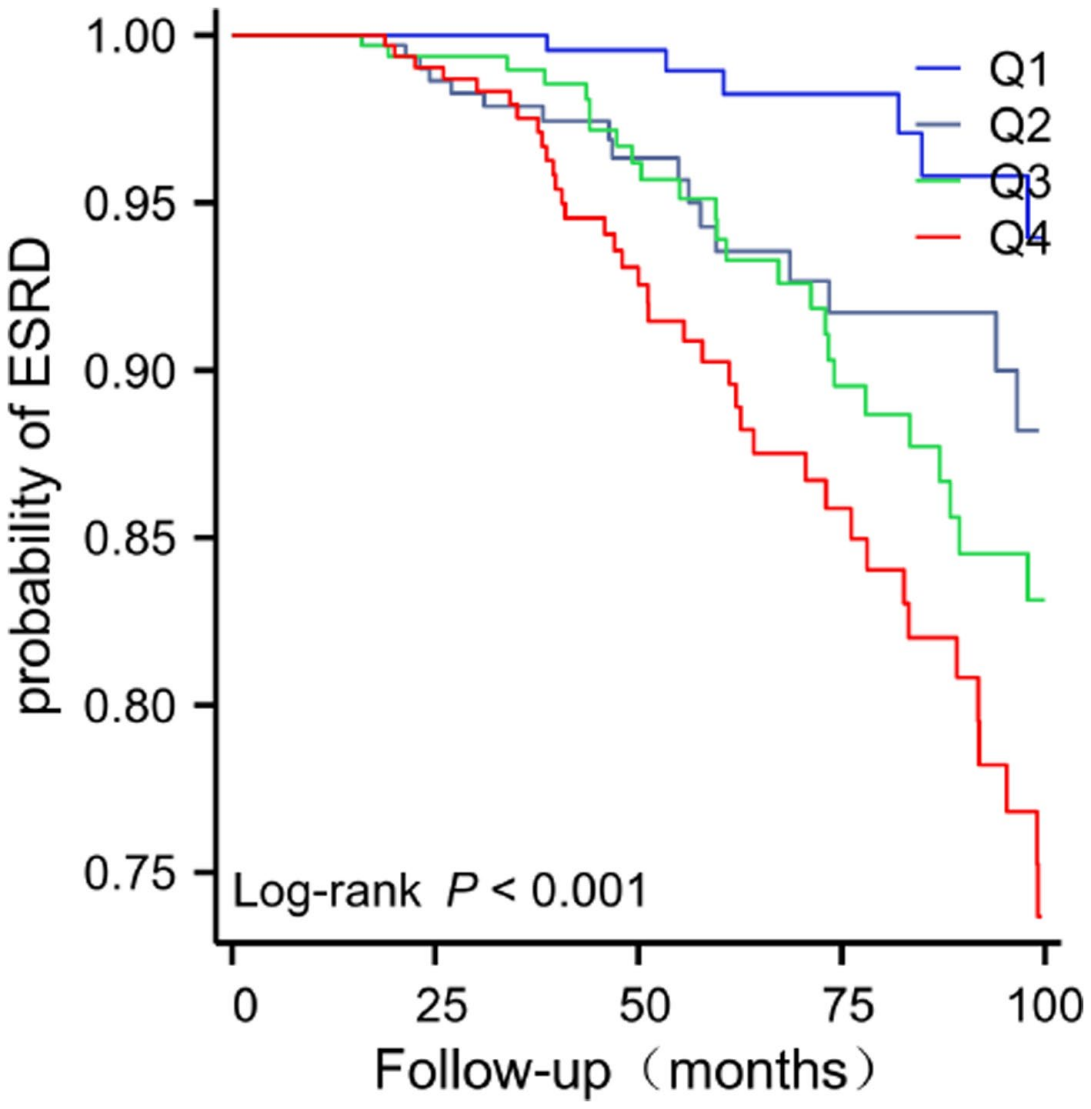
Kaplan–Meier curves showing cumulative ESRD-free survival rates across LDL-C/HDL-C ratio quartiles in 1405 IgAN patients during a median follow-up of 50.1 months. ESRD-free survival rate decreased significantly with increasing LDL-C/HDL-C ratio quartiles (*log-rank p* < 0.001). Abbreviations as in Tables 1 and 2.

During a median follow-up period of 50.1 months, 110 (8.45%) of the 1,405 patients with IgA nephropathy (IgAN) progressed to end-stage renal disease (ESRD). Kaplan–Meier survival analysis (Figure 3) demonstrated that the ESRD-free survival rate declined significantly across increasing quartiles of the LDL/HDL-C ratio, with a statistically significant difference observed among the four quartiles (log-rank *p* < 0.001). The results of univariate and multivariate Cox proportional hazards regression analyses are presented in Table 2. Univariate analysis indicated that, compared with the first quartile (Q1), the second (Q2: HR = 5.816, 95% CI 2.741–12.342, *p* < 0.001), third (Q3: HR = 4.149, 95% CI 1.920–8.968, *p* < 0.001), and fourth (Q4: HR = 3.241, 95% CI 1.442–7.288, *p* = 0.004) quartiles were all associated with a significantly elevated risk of ESRD. Moreover, each 1-unit increase in the LDL-C/HDL-C ratio corresponded to a 50.0% higher ESRD risk (HR = 1.500, 95% CI 1.304–1.726, *p* < 0.001). After adjustment for all potential confounding factors, multivariate Cox regression revealed that the fourth quartile (Q4) remained an independent risk factor for ESRD (HR = 6.384, 95% CI 1.840–22.154, *p* = 0.003), whereas the associations for Q2 (HR = 2.542, 95% CI 0.679–9.523, *p* = 0.166) and Q3 (HR = 2.499, 95% CI 0.707–8.830, *p* = 0.155) were not statistically significant. Additionally, each 1-unit increase in the LDL-C/HDL-C ratio was independently associated with a 45.2% increase in ESRD risk (HR = 1.452, 95% CI 1.159–1.818, *p* = 0.001).

**Table 2:** ESRD risk ratio stratified by baseline LDL-C/HDL-C quartiles.

Characteristics	Unadjusted HR (95% CI)	Unadjusted *p*-value	Multivariable-adjusted HR (95% CI)	Multivariable-adjusted *p*-value
LDL-C/HDL-C quartile				
Q1 (reference)	1.00 (reference)	—	1.00 (reference)	—
Q2	5.816 (2.741–12.342)	<0.001	2.542 (0.679–9.523)	0.166
Q3	4.149 (1.920–8.968)	<0.001	2.499 (0.707–8.830)	0.155
Q4	3.241 (1.442–7.288)	0.004	6.384 (1.840–22.154)	0.003
Per 1-unit increase in LDL-C/HDL-C ratio	1.500 (1.304–1.726)	<0.001	1.452 (1.159–1.818)	0.001

The multivariable model accounted for age, gender, SBP, DBP, sCr, 24 h proteinuria, Alb, hemoglobin, TG, TC, renal pathological classification (M/E/S/T/C), immunoglobulin deposition intensity, and treatment regimens (glucocorticoids, ACEI/ARB, immunosuppressants). HR, hazard ratio; CI, confidence interval; ESRD, end-stage renal disease; other abbreviations are as defined in Table 1.

### 3.3 Linear dose–response relationship between LDL-C/HDL-C ratio and ESRD risk (RCS analysis)

**Figure 4:**
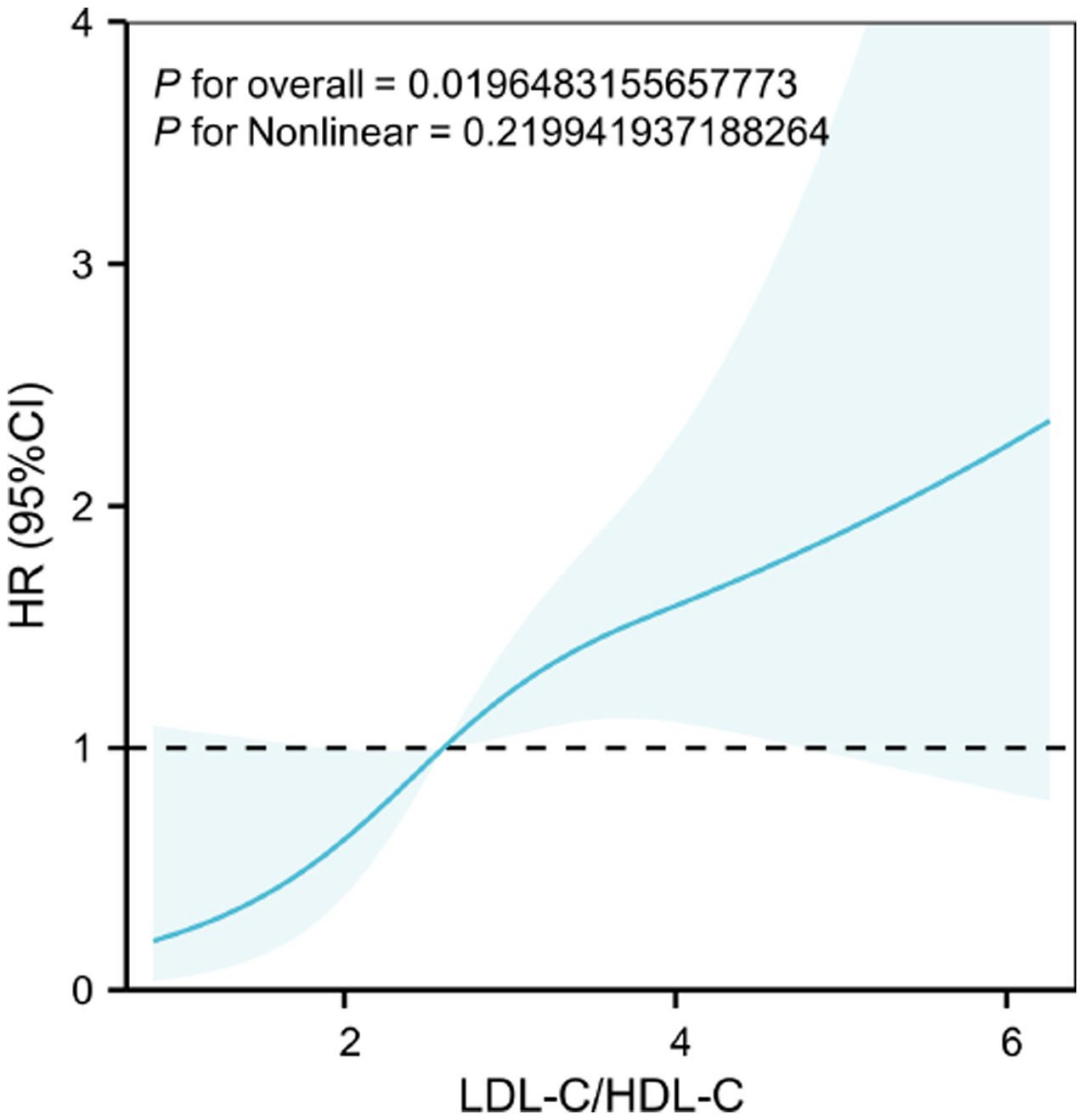
3-knot multivariate-adjusted restricted cubic spline (RCS) plot showing the dose–response relationship between LDL-C/HDL-C ratio and ESRD risk in IgAN patients. The model was adjusted for age, gender, sCr, 24 h proteinuria, SBP, DBP, glucocorticoid use, renal pathological classification, immunoglobulin deposition intensity, Alb, and hemoglobin. The median LDL-C/HDL-C ratio was used as the reference value (HR = 1.0). The shaded area represents the 95% confidence interval (CI). Abbreviations as in Tables 1 and 2.

To further characterize the dose–response relationship between the LDL-C/HDL-C ratio and ESRD risk, we conducted a multivariate-adjusted RCS analysis with three knots, illustrated in Figure 4. This model meticulously adjusted for covariates including age, gender, sCr, urine protein, SBP, DBP, glucocorticoid use, renal pathological classification, immunoglobulin deposition intensity, Alb, and hemoglobin.

The analysis demonstrated a statistically significant overall association between the LDL-C/HDL-C ratio and ESRD risk (*p* for overall = 0.0196). Notably, the test for nonlinearity yielded no statistical significance (*p* for nonlinear = 0.2199), aligning with the distinct linear trend depicted in Figure 4, (which reveals no obvious inflection point. Specifically, as the LDL-C/HDL-C ratio increased, the hazard ratio (HR) for ESRD displayed a progressive linear elevation, confirming a continuous linear dose–response relationship without threshold effects.

### 3.4 Subgroup analysis

**Figure 5:**
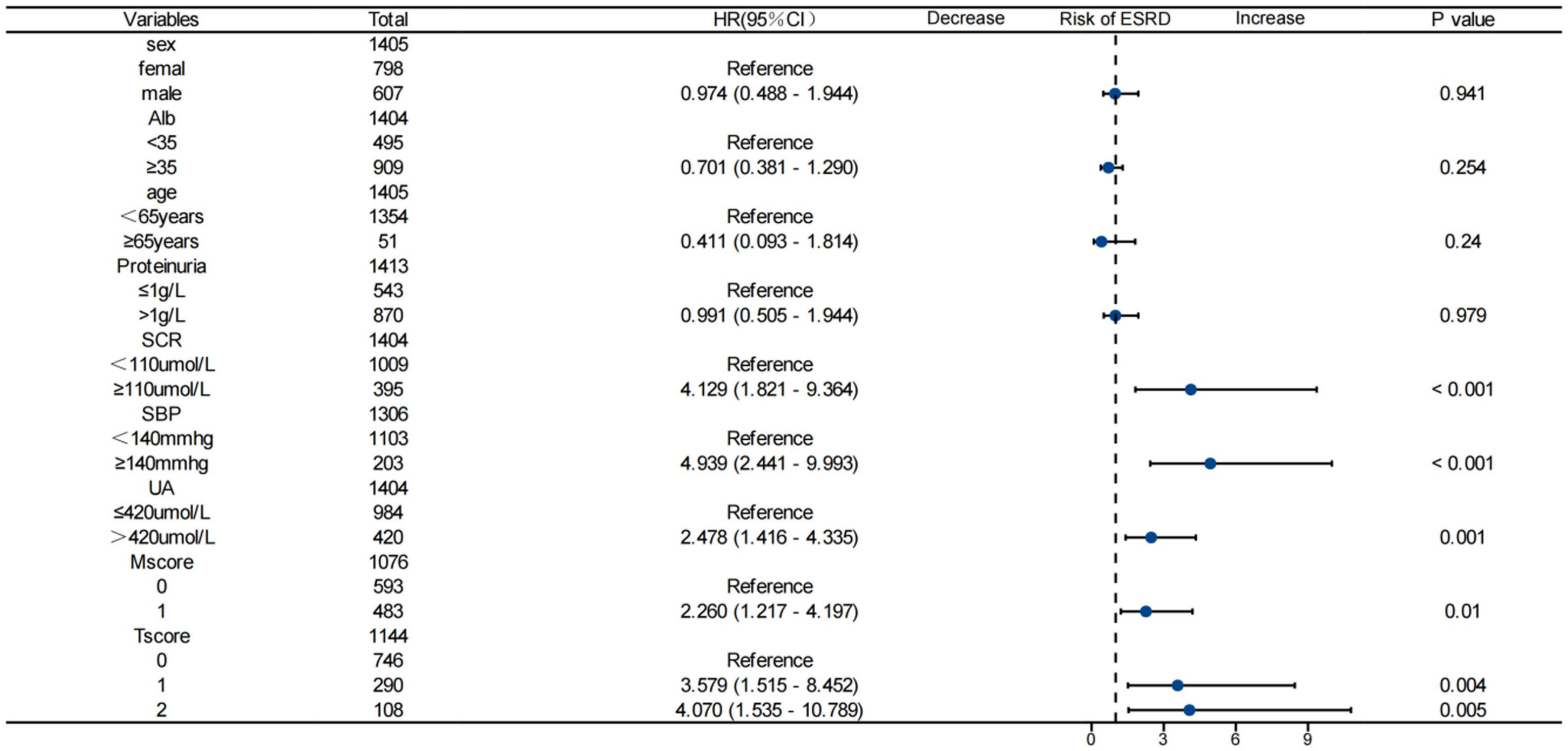
Multivariate-adjusted forest plot showing the association between per 1-unit increase in LDL-C/HDL-C ratio and ESRD risk across different subgroups. The model was adjusted for confounding factors (excluding the subgroup variable itself). Blue hollow circles represent the hazard ratio (HR) point estimate, horizontal lines represent the 95% confidence interval (CI), and red solid circles indicate statistically significant associations (*p* < 0.05). Abbreviations as in Tables 1 and 2.

Subgroup analysis (Figure 5) was performed to explore the consistency of the association between the LDL-C/HDL-C ratio (continuous variable) and ESRD risk across different subgroups stratified by baseline clinical and pathological characteristics. The results showed that the positive association between the LDL-C/HDL-C ratio and ESRD risk was significantly amplified in the subgroups of patients with elevated sCr, hypertension, hyperuricemia, mesangial cell proliferation (M1), and severe tubular atrophy/interstitial fibrosis (T2) (all *p* < 0.05). No significant interaction was observed between the LDL-C/HDL-C ratio and other baseline characteristics (age, gender, E classification, S classification, C classification, glucocorticoid use, ACEI/ARB use) with respect to ESRD risk (all *p* for interaction >0.05), indicating that the prognostic value of the LDL-C/HDL-C ratio was consistent across these subgroups.

## 4 Discussion

This bicenter retrospective cohort study potentially demonstrates for the first time that an elevated LDL-C/HDL-C ratio serves as an independent risk factor for ESRD in IgAN patients, establishing a significant linear dose–response relationship between this ratio and ESRD risk through RCS analysis. Additionally, we found that the prognostic value of LDL-C/HDL-C ratio is amplified in IgAN patients with hypertension, hyperuricemia, mesangial proliferation, and severe tubulointerstitial fibrosis, and crucially, LDL-C/HDL-C ratio is not associated with the core immunological characteristic of IgAN (renal IgA deposition intensity). These findings suggest that LDL-C/HDL-C ratio can serve as a novel lipid biomarker for prognostic stratification of IgAN patients, and lipid metabolism abnormalities may be an independent pathological process in IgAN progression, independent of the immunological abnormality of IgA deposition.

### 4.1 LDL-C/HDL-C ratio as an independent prognostic biomarker for IgAN

Single lipid parameters including LDL-C and HDL-C have been previously linked to the prognostic outcomes of IgAN (4, 6), yet the present study further demonstrates that the combined LDL-C/HDL-C ratio harbors more stable and reliable prognostic value for this disease. Multivariate Cox regression analysis revealed that after adjusting for a comprehensive set of clinical and pathological confounding factors, patients in the fourth quartile (Q4) of the LDL-C/HDL-C ratio had a 6.384-fold higher risk of developing ESRD compared with those in the first quartile (Q1), and each 1-unit increase in the LDL-C/HDL-C ratio was independently associated with a 45.2% elevated ESRD risk. This finding is consistent with prior studies in the general population and other chronic kidney disease (CKD) subtypes, which have identified the LDL-C/HDL-C ratio as a superior predictor of renal dysfunction relative to individual lipid indicators (18, 19). The reason may be that the LDL-C/HDL-C ratio can comprehensively reflect the balance between pro-atherosclerotic (LDL-C) and anti-atherosclerotic (HDL-C) lipid factors, and its change can more accurately reflect the degree of lipid metabolism disorder than a single indicator (8, 9). In addition, our study found that the ESRD risk increased only in Q4 of the LDL-C/HDL-C ratio, suggesting that there may be a threshold effect of the LDL-C/HDL-C ratio on the prognosis of IgAN, and aggressive lipid-lowering intervention may be needed for patients with a high LDL-C/HDL-C ratio (Q4) to reduce ESRD risk.

### 4.2 Linear dose–response relationship between LDL-C/HDL-C ratio and ESRD risk

Our 3-knot RCS analysis confirmed a linear association between the LDL-C/HDL-C ratio and ESRD risk in IgAN patients (nonlinear *p* = 0.2199), a finding that differs from some previous investigations into lipid parameters and CKD prognosis (5–7). This linear dose–response relationship indicates that for every 1-unit increase in the LDL-C/HDL-C ratio, the ESRD risk in IgAN patients rises by a fixed 45.2%, and there is no apparent “safe range” for this ratio in the context of IgAN prognosis. This finding has important clinical implications: in the clinical management of IgAN patients, it is necessary to monitor the LDL-C/HDL-C ratio regularly, and maintain it at a low level as much as possible to delay kidney function decline, rather than just focusing on whether it exceeds a certain threshold.

### 4.3 Amplified prognostic value in high-risk IgAN subgroups

Subgroup analysis further elucidated that the positive association between the LDL-C/HDL-C ratio and ESRD risk is significantly amplified in IgAN patients with specific high-risk clinical and pathological characteristics, including hypertension, hyperuricemia, mesangial cell proliferation (M1), and severe tubular atrophy/interstitial fibrosis (T2). This amplified association is likely attributable to the synergistic pathological effects between lipid metabolism disorders and these high-risk factors: (1) Hypertension is closely associated with RAAS activation, and animal studies have confirmed that RAAS activation can mediate lipid abnormality-induced renal injury (20); (2) Hyperuricemia can suppress nitric oxide synthesis, impair insulin regulation of plasma free fatty acids, stimulate hepatic overproduction of LDL-C, and promote HDL-C excretion, further increasing the LDL-C/HDL-C ratio (21), and urate deposition can cause renal interstitial inflammation and fibrosis, which synergizes with lipid toxicity to accelerate kidney function decline; (3) Mesangial cell proliferation and tubulointerstitial fibrosis are the core pathological features of IgAN progression (22–26), and LDL-C can directly promote mesangial cell proliferation and extracellular matrix accumulation (27, 28), while oxidized LDL (OX-LDL) can induce tubular epithelial cell dysfunction and apoptosis (29, 30), thus amplifying the pathological damage of lipid metabolism abnormalities in patients with these pathological changes. These findings suggest that IgAN patients with both a high LDL-C/HDL-C ratio and the above high-risk factors are the key population for clinical intervention, and combined intervention targeting lipid metabolism, blood pressure, uric acid, and pathological damage is needed to improve their prognosis.

### 4.4 Independence of LDL-C/HDL-C ratio from renal IgA deposition

Renal IgA deposition is the core and specific immunological hallmark of IgAN (2), as the disease is pathologically defined by dominant mesangial IgA deposition—this feature distinguishes IgAN from other glomerular diseases (9). Clarifying the relationship between prognostic biomarkers and IgA deposition is critical for elucidating the underlying pathological mechanisms of disease progression. Spearman correlation analysis in the present study confirmed no significant correlation between the LDL-C/HDL-C ratio and the intensity of renal IgA deposition in IgAN patients (*r* = 0.013, *p* = 0.624), indicating that the prognostic value of the LDL-C/HDL-C ratio is independent of the core immunological abnormality of IgAN.

To ensure research rigor and avoid selective reporting, we also analyzed the association between the ratio and renal IgG, IgM deposition. Although IgG deposition intensity differed among ratio quartiles (*p* < 0.001), there was no monotonic correlation with the ratio itself (*r* = −0.039, *p* = 0.145), suggesting this difference was an accidental group distribution variation rather than a ratio-driven change. IgM deposition showed no interquartile difference (*p* = 0.743) and no correlation with the ratio (*r* = −0.022, *p* = 0.405). We focused on IgA in the abstract and core discussion because IgA deposition is IgAN-specific, while IgG/IgM deposition are common in multiple glomerular diseases and lack disease-specificity (12). Importantly, multivariate Cox regression has adjusted for the intensity of all immunoglobulin depositions (including IgG and IgM), and the ratio remained an independent risk factor for ESRD—confirming that IgG/IgM deposition does not interfere with the core conclusion.

This key finding supports the notion that dyslipidemia constitutes an independent pathological process in IgAN progression, which is not mediated by the classical IgA immunological pathway. Potential pathological mechanisms underlying the LDL-C/HDL-C ratio-driven IgAN progression include the following: (1) A high LDL-C/HDL-C ratio is linked to atherosclerosis, with LDL-C inducing glomerulosclerosis by promoting endothelial and mesangial cell proliferation (27, 31); (2) Oxidized LDL (OX-LDL), derived from LDL-C oxidation in hypercholesterolemia, exerts cytotoxic effects on glomerular podocytes and tubular epithelial cells (28, 29); (3) OX-LDL stimulates monocytes/macrophages to secrete pro-inflammatory cytokines and chemokines, accelerating renal interstitial inflammation (30, 32); (4) Reduced HDL-C levels (a key contributor to an elevated LDL-C/HDL-C ratio) impair its anti-inflammatory and antioxidant protective effects on renal parenchymal cells (33), and the abnormal quantity and quality of HDL in CKD patients further exacerbate such lipid-mediated renal injury (34). Collectively, this dual lipid impairment (elevated LDL-C, reduced functional HDL-C) forms a non-immunological pathological axis that accelerates IgAN progression independent of IgA deposition.

### 4.5 Clinical implications

This study provides key clinical insights for IgAN management: (1) The LDL-C/HDL-C ratio, a routine and reproducible laboratory indicator, should be incorporated into IgAN prognostic stratification systems for reliable ESRD risk assessment; (2) Individualized lipid-lowering interventions are warranted for patients with high LDL-C/HDL-C ratios (especially Q4), with therapeutic targets focused on maintaining LDL-C/HDL-C balance rather than just LDL-C reduction; (3) Combined multi-factor intervention is essential for high-risk patients with concurrent elevated LDL-C/HDL-C ratio, hypertension, hyperuricemia or severe renal pathology, to maximize ESRD risk reduction.

### 4.6 Limitations and future directions

This study is a retrospective bicenter design, which may introduce selection bias. Although we adjusted for multiple confounding factors, residual confounding cannot be completely excluded. Second, we only measured baseline LDL-C/HDL-C ratio and did not monitor its dynamic changes during follow-up, which limits the exploration of the impact of ratio fluctuations on prognosis. Third, the sample size, while relatively large, is still from two centers in China, and the results may need validation in multi-center, multi-ethnic cohorts. Future prospective studies are warranted to verify the findings and explore the effect of lipid-lowering interventions targeting LDL-C/HDL-C ratio on IgAN prognosis.”

## 5 Conclusion

An elevated LDL-C/HDL-C ratio is an independent risk factor for ESRD in IgAN patients, with a significant linear dose–response relationship to ESRD risk. Its prognostic value is notably amplified in high-risk subgroups (hypertension, hyperuricemia, M1, T2) and is independent of renal IgA deposition intensity—suggesting lipid metabolism disorder is an independent pathological process in IgAN progression. Collectively, the LDL-C/HDL-C ratio is a novel, convenient, and reliable lipid biomarker for ESRD risk stratification in IgAN, and lipid-lowering interventions targeting this ratio may confer significant clinical benefits to high-risk IgAN patients.

## Data Availability

The raw data supporting the conclusions of this article will be made available by the authors, without undue reservation.

## References

[1] Rodrigues JC Haas M Reich HN. IgA nephropathy. Clin J Am Soc Nephrol. (2017) 12:677–86. doi: 10.2215/CJN.08680816, 28159829 PMC5383386

[2] Suzuki H Kiryluk K Novak J Moldoveanu Z Herr AB Renfrow MB . The pathophysiology of IgA nephropathy. J Am Soc Nephrol. (2011) 22:1795–803. doi: 10.1681/ASN.2010080881, 21949093 PMC3892742

[3] Florens N Calzada C Lyasko E Juillard L Soulage CO. Modified lipids and lipoproteins in chronic kidney disease: a new class of uremic toxins. Toxins. (2016) 8:376. doi: 10.3390/toxins8120376, 27999257 PMC5198570

[4] Poss J Custodis F Werner C Weingartner O Bohm M Laufs U. Cardiovascular disease and dyslipidemia: beyond LDL. Curr Pharm Des. (2011) 17:861–70. doi: 10.2174/138161211795395367 21418031

[5] Tian ZY Li AM Chu L Hu J Xie X Zhang H. Prognostic value of low-density lipoprotein cholesterol in IgA nephropathy and establishment of nomogram model. Front Endocrinol. (2023) 14:1037773. doi: 10.3389/fendo.2023.1037773, 36843611 PMC9950098

[6] Zhong J Yang H Kon V. Kidney as modulator and target of “good/bad” HDL. Pediatr Nephrol. (2019) 34:1683–95. doi: 10.1007/s00467-019-04276-8, 30291429 PMC6450786

[7] Baragetti A Norata GD Sarcina C Rastelli F Grigore L Garlaschelli K . High density lipoprotein cholesterol levels are an independent predictor of the progression of chronic kidney disease. J Intern Med. (2013) 274:252–62. doi: 10.1111/joim.12093, 23607805

[8] Sun T Chen M Shen H PingYin Fan L Chen X . Predictive value of LDL/HDL ratio in coronary atherosclerotic heart disease. BMC Cardiovasc Disord. (2022) 22:273. doi: 10.1186/s12872-022-02706-6, 35715736 PMC9206383

[9] Trimarchi H Barratt J Cattran DC Cook HT Coppo R Haas M . Oxford classification of IgA nephropathy 2016: an update from the IgA nephropathy classification working group. Kidney Int. (2017) 91:1014–21. doi: 10.1016/j.kint.2017.02.003, 28341274

[10] Ntimana CB Mashaba RG Seakamela KP Mphekgwana PM Nemuramba R Mothapo K . Association between renal dysfunction and lipid ratios in rural black south Africans. Int J Environ Res Public Health. (2025) 22:324. doi: 10.3390/ijerph22030324, 40238327 PMC11942230

[11] Levey AS Stevens LA Schmid CH Zhang Y Castro AF III Feldman HI . CKD-EPI creatinine equation: a new equation to estimate glomerular filtration rate from serum creatinine in adults. Ann Intern Med. (2009) 150:604–12. doi: 10.7326/0003-44819-100905050-00007, 19414839 PMC2763564

[12] Mori K Nihei Y Suzuki H Suzuki Y. Identification of the localization of the pathogenic IgA autoantibodies on mesangial cells in IgA nephropathy. J Am Soc Nephrol. (2024) 35. doi: 10.1681/ASN.2024cnmrqr3c, 28159829

[13] Sedgwick P. Spearman’s rank correlation coefficient. BMJ. (2014) 349:g7327. doi: 10.1136/bmj.g7327 25432873

[14] Kaplan EL Meier P. Nonparametric estimation from incomplete observations. J Am Stat Assoc. (1958) 53:457–81. doi: 10.1080/01621459.1958.10501452

[15] Cox DR. Regression models and life-tables. J R Stat Soc Ser B Methodol. (1972) 34:187–220. doi: 10.1111/j.2517-6161.1972.tb00899.x

[16] Harrell FE Jr Lee KL Mark DB. Multivariable prognostic models: issues in developing models, evaluating assumptions and adequacy, and measuring and reducing errors. Stat Med. (1996) 15:361–87. doi: 10.1002/(SICI)1097-0258(19960229)15:4<361::AID-SIM168>3.0.CO;2-4, 8668867

[17] Altman DG Bland JM. Interaction revisited: the difference between two estimates. BMJ. (2003) 326:219. doi: 10.1136/bmj.326.7382.219, 12543843 PMC1125071

[18] Navise NH Mokwatsi GG Gafane-Matemane LF Fabian J Lammertyn L. Kidney dysfunction: prevalence and associated risk factors in a community-based study from the north west province of South Africa. BMC Nephrol. (2023) 24:23. doi: 10.1186/s12882-023-03068-7, 36717778 PMC9887915

[19] Kim JY Kang HT Lee HR Lee YJ Shim JY. Comparison of lipid-related ratios for prediction of chronic kidney disease stage 3 or more in Korean adults. J Korean Med Sci. (2012) 27:1524–9. doi: 10.3346/jkms.2012.27.12.1524, 23255852 PMC3524432

[20] Ni J Ma KL Wang CX Liu J Zhang Y Lv LL . Activation of renin-angiotensin system is involved in dyslipidemia-mediated renal injuries in apolipoprotein E knockout mice and HK-2 cells. Lipids Health Dis. (2013) 12:49. doi: 10.1186/1476-511X-12-49, 23570453 PMC3706287

[21] Choi HK Ford ES. Prevalence of the metabolic syndrome in individuals with hyperuricemia. Am J Med. (2007) 120:442–7. doi: 10.1016/j.amjmed.2006.06.040 17466656

[22] Reich HN Troyanov S Scholey JW Cattran DC Toronto Glomerulonephritis Registry. Remission of proteinuria improves prognosis in IgA nephropathy. J Am Soc Nephrol. (2007) 18:3177–83. doi: 10.1681/ASN.2007050526 17978307

[23] Goto M Wakai K Kawamura T Ando M Endoh M Tomino Y. A scoring system to predict renal outcome in IgA nephropathy: a nationwide 10-year prospective cohort study. Nephrol Dial Transplant. (2009) 24:3068–74. doi: 10.1093/ndt/gfp273, 19515800 PMC2747499

[24] Kawamura T Joh K Okonogi H Koike K Utsunomiya Y Miyazaki Y . A histologic classification of IgA nephropathy for predicting long-term prognosis: emphasis on end-stage renal disease. J Nephrol. (2013) 26:350–7. doi: 10.5301/jn.5000151, 22684645

[25] Howie AJ Lalayiannis AD. Systematic review of the Oxford classification of IgA nephropathy: reproducibility and prognostic value. Kidney360. (2023) 4:1103–11. doi: 10.34067/KID.0000000000000195, 37357346 PMC10476683

[26] Zhu X Li H Liu Y You J Qu Z Yuan S . Tubular atrophy/interstitial fibrosis scores of Oxford classification combined with proteinuria level at biopsy provides earlier risk prediction in IgA nephropathy. Sci Rep. (2017) 7:1100. doi: 10.1038/s41598-017-01223-3, 28439112 PMC5430886

[27] Joles JA van Goor H van der Horst M van Tol A Elema JD Koomans HA. High lipid levels in very low density lipoprotein and intermediate density lipoprotein may cause proteinuria and glomerulosclerosis in aging female analbuminemic rats. Lab Investig. (1995) 73:912–21. doi: 10.1038/labinvest.1995.146, 8558854

[28] Ferro CJ Mark PB Kanbay M Sarafidis P Heine GH Rossignol P . Lipid management in patients with chronic kidney disease. Nat Rev Nephrol. (2018) 14:727–49. doi: 10.1038/s41581-018-0072-9 30361677

[29] Gyebi L Soltani Z Reisin E. Lipid nephrotoxicity: new concept for an old disease. Curr Hypertens Rep. (2012) 14:177–81. doi: 10.1007/s11906-012-0250-2, 22290079

[30] Glass CK Witztum JL. Atherosclerosis: the road ahead. Cell. (2001) 104:503–16. doi: 10.1016/s0092-8674(01)00238-0 11239408

[31] Nishida Y Oda H Yorioka N. Effect of lipoproteins on mesangial cell proliferation. Kidney Int Suppl. (1999) 56:S51–3. doi: 10.1046/j.1523-1755.1999.07113.x 10412737

[32] Vaziri ND. Role of dyslipidemia in impairment of energy metabolism, oxidative stress, inflammation and cardiovascular disease in chronic kidney disease. Clin Exp Nephrol. (2014) 18:265–8. doi: 10.1007/s10157-013-0847-z, 23974528

[33] Thiemermann C Patel NSA Kvale EO Cockerill GW Brown PAJ Stewart KN . High density lipoprotein (HDL) reduces renal ischemia/reperfusion injury. J Am Soc Nephrol. (2003) 14:1833–43. doi: 10.1097/01.asn.0000075552.97794.8c, 12819243

[34] Attman PO Samuelsson O Alaupovic P. Lipoprotein metabolism and renal failure. Kidney Int. (1999) 56:42–6. doi: 10.1046/j.1523-1755.1999.07111.x 8503411

